# Bile Derivative T3K Ameliorates Colitis by Regulating the Intestinal Microbiota-Bile Acid Axis

**DOI:** 10.3390/pharmaceutics18010020

**Published:** 2025-12-23

**Authors:** Yu Zhou, Yixiang Zhang, Ying Li, Yu Chen, Xiaoqian Chi, Zhongyu You, Haijing Zhang, Yong Li, Lianqiu Wu

**Affiliations:** 1 State Key Laboratory of Digestive Health, Institute of Materia Medica, Chinese Academy of Medical Sciences & Peking Union Medical College, Beijing 100050, China; zhou.yu@imm.ac.cn (Y.Z.); zhangyxx0119@163.com (Y.Z.); chen.yu@imm.ac.cn (Y.C.); cxqneon@gmail.com (X.C.); youzhongyu@imm.ac.cn (Z.Y.); wlq@imm.ac.cn (L.W.); 2 State Key Laboratory of Bioactive Substance and Function of Natural Medicines, Institute of Materia Medica, Chinese Academy of Medical Sciences & Peking Union Medical College, Beijing 100050, China; liyingly@imm.ac.cn

**Keywords:** ulcerative colitis, bile acid, pseudo-germ-free, *Akkermansia muciniphila*, muricholic acid

## Abstract

**Background/Objectives**: The pathogenesis of ulcerative colitis (UC) is complex, and there is an urgent need for effective therapeutic agents with low side effects. Recent studies highlight the critical roles of abnormal bile acid (BA) metabolism and gut microbiota dysbiosis in UC progression. However, there is a significant knowledge gap about the relation between BA and gut microbiota. The BA derivative T3K exerts good anti-UC effect, and its mechanism is still unknown. In this study, we investigate how its anti-UC mechanism is involved in the modulation of the gut microbiota-BA axis and BA metabolism. **Methods**: Gene expression microarray GSE92415 of UC from the Gene Expression Omnibus was used to analyze BA metabolism. DSS-induced colitis mouse model, Caco-2 and IEC6 cells were used to confirm the anti-UC of T3K using intestinal permeability assay with FITC, Western-blot, immunohistochemical staining, immunofluorescenc and so on in vitro and in vivo. The changes in bile acid and microbiota were measured by 16S rRNA sequencing and bile acid analysis combined with pseudo-germ-free (PGF) models and fecal microbiota transplantation (FMT). **Results**: T3K demonstrated strong therapeutic effects, including reduced weight loss, lower disease activity index (DAI), and increased colon length. T3K also enhanced the expression of Occludin and Mucin2, and restored gut barrier integrity. Furthermore, T3K improved intestinal dysbiosis and abnormal BA metabolism in colitis mice. Through PGF models and FMT, we confirmed that T3K modulates BA metabolism via the gut microbiota. T3K specifically promotes the growth of beneficial bacteria, such as *Akkermansia muciniphila*, increases levels of hydrophilic BAs like muricholic acid (MCA), lithocholic acid (LCA) and its derivatives isoLCA and then repairs damaged intestinal mucosa. **Conclusions**: Bile acid derivative T3K, as a potential anti-UC candidate, effectively restores gut barrier integrity and then ameliorates colitis by improving gut microbiota composition and regulating BA metabolism, including increasing hydrophilic BAs.

## 1. Introduction

Ulcerative colitis (UC) is a chronic inflammatory disease of unknown cause that primarily affects the colonic mucosa, starting in the rectum and often spreading continuously throughout the colon [1]. Its prevalence has remained high in developed countries, with increasing rates in developing countries since the early 20th century, making it a significant global health and economic burden [2,3]. Current treatments—5-aminosalicylic acid, thiopurines, biologics (anti-cytokines, anti-integrins), small-molecule drugs, and probiotics—are only moderately effective, with remission rates of 30% to 60%. The multifactorial pathogenesis of UC, involving genetic, environmental, microbial, and immune interactions, has limited progress in developing more effective therapies [4].

The intestinal microbiota is essential for health and plays a key role in disease. In healthy individuals, it comprises about 1000 to 2000 microbial species, but in inflammatory bowel disease (IBD), including UC, microbial diversity is significantly reduced, resulting in dysbiosis [5,6,7]. IBD patients exhibit an increased presence of Proteobacteria and Fusobacteria, including *Enterobacteriaceae*, *Escherichia coli*, *Klebsiella pneumoniae*, *Pasteurellaceae*, *Neisseriaceae*, and *Fusobacterium varium*, while beneficial species such as *Clostridiales*, *F. prausnitzii*, *E. rectalis*, and *Firmicutes* are diminished [5,6,7,8,9,10,11]. Additionally, specific metabolites produced by gut microbes, such as bile acids (BAs), short-chain fatty acids, and tryptophan derivatives, are implicated in IBD pathogenesis [12,13]. BAs, synthesized from cholesterol in the liver, play a key role in the absorption of fats, fat-soluble vitamins, and nutrients in the intestines due to their amphipathic properties. Gut microbiota convert primary BAs into secondary forms, with microbial composition influencing BA diversity. Recent research has revealed their additional functions as regulators of glucose and lipid metabolism, energy homeostasis, and immune response [11].

In traditional Chinese medicine, bear bile is prized for its anti-inflammatory properties and clinical benefits [14]. It has been shown to alleviate UC symptoms in murine models, attracting significant scientific interest [15]. Bear bile includes primary bile acids (CA, CDCA), secondary bile acids (UDCA, DCA, LCA), and conjugated bile acids (GUDCA, TUDCA, TCDCA). Our research further investigates its potential in UC treatment, focusing on the effects of tauroursodeoxycholate (TUDCA) and taurochenodeoxycholate (TCDCA), though their exact mechanisms remain unclear. Additionally, we have identified tauro-7α-hydroxy-3-oxo-5β-cholanoate (T3KDCA, abbreviated as T3K), which makes up about 3.1% of bear bile [16]. Its free acid form, 7α-hydroxy-3-oxo-5β-cholanoate (3KDCA, abbreviated as 3K), is also an endogenous secondary BA in humans [17]. While TUDCA has shown anti-UC effects and is under clinical evaluation for safety and efficacy [18], T3K has demonstrated even greater therapeutic potential, warranting deeper investigation. This study employs a murine colitis model, along with pseudo-germ-free (PGF) conditions and fecal microbiota transplantation (FMT), to thoroughly assess T3K’s anti-UC efficacy and its underlying mechanisms.

## 2. Materials and Methods

### 2.1. Compounds

TUDCA, TCDCA and T3K were synthesized by our research team (structures are depicted in Supplemental Figure S1A) [16]. A concise synthesis route for T3K is illustrated in Supplemental Figure S1B. The purity of the compounds was all above 98%. Drained bear bile powder (XDF) was provided by China National Traditional Chinese Medicine Corporation (Beijing, China). Sulfasalazine (SASP) was purchased from Shanghai sphsine Co., Ltd. (Shanghai, China).

### 2.2. Animals and Cells

C57BL/6J (male, 20–22 g) were purchased from Beijing Huafukang Biotechnology Co., Ltd. (Beijing, China), and raised in the Animal House of Institute of Materia Medica, Chinese Academy of Medical Sciences. The animal feeding environment was maintained at a temperature of 23–25 °C, with a humidity level of 40–60% and a light/dark cycle of 12 h. The animals were provided with free access to food and water at an adaptation.

Caco-2 cells (Wuhan Punosai Life Technology Co., Ltd., Wuhan, China) were cultured in 1640 medium with 10% FBS and 100 U/mL penicillin–streptomycin solution at 37 °C under 5% CO_2_. IEC6 cells were obtained from the Institute of Basic Medical Sciences, Chinese Academy of Medical Sciences (Beijing, China), and cultured in DMEM with 10% FBS and 100 U/mL penicillin–streptomycin solution at 37 °C under 5% CO_2_.

*Akkermansia muciniphila* powder was obtained from Shanghai Xinyu Biotechnology Co., Ltd. (Shanghai, China). The powder was dissolved in the liquid thioglycolate medium and transferred into a sealed culture bag along with AnaeroPack(MGC, Mitsubishi, Tokyo, Japan) and oxygen indicator, before being placed in an incubator at 37 °C for co-cultivation.

### 2.3. Bioinformatics Database Search and Analysis

We obtained the gene expression profiling microarray GSE92415 of UC from the Gene Expression Omnibus (GEO, https://www.ncbi.nlm.nih.gov/geo/, accessed on 23 July 2023) database using “GEOquery” R package (v3.6.0). The GSE92415 contains 87 UC patients, 21 healthy volunteers and 75 treated patients, and we removed the treated patient data. Then KEGG analysis and genes involved in BA metabolism were, respectively, analyzed. The specific methodological details are as follows:

Differential Expression Gene (DEG) Identification: We employed the limma package in R to fit linear models based on an empirical Bayesian approach for DEG identification. We set the statistical threshold for genes to be considered differentially expressed as: |log_2_FC| > 1 and *p*-value < 0.05; KEGG pathway enrichment analysis: Following the identification of DEGs, we performed KEGG pathway enrichment analysis on the DEGs using the clusterProfiler package in R. We employed a hypergeometric distribution test to determine whether differentially expressed genes were significantly enriched in specific biological pathways. The enrichment analysis was based on the KEGG database (updated October 2023), with *p*-value < 0.05. Definition Criteria for Bile Acid-Related Genes: For analyzing bile acid metabolism-related genes, we defined the target gene set by integrating authoritative database resources. We extracted all genes from the KEGG pathway database associated with the human primary bile acid biosynthesis pathway (hsa00120) and the secondary bile acid biosynthesis pathway (hsa00121). Additionally, we supplemented this with the GeneCards database, searching using the keyword “bile acid metabolism” and selecting genes with an association score above 10. Merging genes from both sources formed a comprehensive reference list of bile acid metabolism-related genes.

### 2.4. Establishment of DSS-Induced Colitis Mouse Model

After acclimatization for 7 days, the animals were randomly distributed into the control group (CON), model group (MOD), administration groups (SASP, T3K, TUDCA, TCDCA, XDF). Each group consisted of 6 mice, totaling 42 mice. Except for the CON group, other groups were induced by 3% (*w*/*v*) DSS in drinking water for 7 days, with the solution prepared and replaced every other day. The mice were euthanized on day 8 and the entire colon was measured. The results section includes body weight changes, Disease activity index (DAI), colon length measurements, and all sample collections, all of which were measured and obtained on the day of mouse euthanasia. DAI was carried out as described previously [19]. A segment of the distal colon measuring 1 cm was collected for histological staining, and the remaining colon was snap-frozen in −80 °C. Feces samples were collected for 16S rRNA analysis and bile acid analysis.

### 2.5. Establishment of DSS-Induced PGF Colitis Mouse Model

After 7 days of acclimation, the PGF colitis mouse model was established by administering a cocktail of antibiotics (vancomycin 0.1 g/L, ampicillin 0.2 g/L, metronidazole 0.2 g/L, and neomycin sulfate 0.2 g/L for 4 days) before DSS induction to deplete the gut microbiota. Then the PGF mice were randomly distributed into the PGF-control (PGF-CON), PGF-model (PGF-MOD), PGF-model + T3K (PGF-T3K, 500 mg/kg) groups. Each group consisted of 6 mice, totaling 18 mice. Except for the PGF-CON group, other groups were induced by 3% (*w*/*v*) DSS in drinking water for 9 days, with the solution prepared and replaced every other day. The mice were euthanized on day 13, and the entire colon was measured. The results section includes body weight changes, DAI values, colon length measurements, and all sample collections, all of which were measured and obtained on the day of mouse euthanasia.

### 2.6. Establishment of FMT DSS-Induced PGF Colitis Mouse Model

FMT was performed as previously described with necessary modifications to suit the experimental conditions [20,21,22]. C57BL/6J mice were acclimated for one week in an SPF-grade animal facility before undergoing the following experimental design: Fecal Microbiota Transplant Experimental Donor Group (Donor-MOD group), T3K-Administered Donor Group (Donor-T3K group), control Group (CON group), Model Group (MOD group), FMT Model Recipient Group (FMT-MOD group), FMT-T3K Recipient Group (FMT-T3K Group). Groups were randomly assigned with 8 mice per group, totaling 48 mice. The Donor-MOD Group received distilled water (0.2 mL/20 g) daily, while the Donor-T3K Group received T3K solution (500 mg/kg, 0.2 mL/20 g) via gavage every other day for two weeks. Normal drinking water was replaced with 3% (*w*/*v*) DSS solution for 7 days. Fresh feces from donor group mice were collected one day prior to fecal microbiota transplantation and stored at −80 °C. Recipient mice received a free-drinking access to a quadruple antibiotic mixture for 4 days to clear intestinal bacteria. Following antibiotic treatment, fecal microbiota transplantation was performed every other day for the subsequent two weeks, totaling 7 transplants. For each FMT, 300 mg of fresh fecal matter from donor mice was suspended in 3 mL PBS and administered to recipient mice via gavage at a volume of 0.2 mL/20 g body weight every other day. On day 11 of the experiment, the drinking water for the MOD group, FMT-MOD group, and FMT-T3K group was replaced with a 3% (*w*/*v*) DSS solution for free access for 7 days, with the solution prepared and replaced every other day. The CON group and MOD group received distilled water via gavage at a volume of 0.2 mL/20 g body weight, starting from the day after modeling and continuing until the end of the experiment. The results section includes body weight changes, DAI, colon length measurements, and all sample collections, all of which were measured and obtained on the day of mouse euthanasia.

### 2.7. Intestinal Permeability Assay

Intestinal permeability was measured using fluorescein isothiocyanate (FITC)-dextran as described previously [23]. The mice were deprived of food but not water overnight. Mice were gavaged with FITC-dextran at a dosage of 800 mg/kg. After 4 h, blood was collected and the serum was separated. The concentration of FITC in serum was measured with an excitation of 485 nm and an emission wavelength of 528 nm.

### 2.8. Histopathological Examination

Colon tissues were fixed in 4% paraformaldehyde, embedded in paraffin, and subsequently stained with hematoxylin and eosin (H&E) for histological examination. Histological analysis was conducted based on the criteria established by Dieleman LA et al. [24].

### 2.9. 16S rRNA Sequencing Analysis

A 16S rRNA sequencing analysis was conducted by TinyGen Biotechnology (Shanghai, China) Co., Ltd. The total DNA of fecal genome was extracted using the QIAamp DNA Stool Mini Kit (Univ, Shanghai, China). Detailed information regarding the bioinformatics analysis workflow is provided in the Supplementary Materials. The V4–V5 hypervariable regions of the 16S rRNA gene were amplified using genomic DNA from fecal as the template and primers (515F 5′-GTGCCAGCMGCCGCGGTAA-3′; 926R 5′-CCGTCAATTCMTTTGAGTTT-3′) targeting conserved bacteria sequences were employed. Subsequently, the PCR amplification products were subjected to sequencing on an Illumina MiSeq platform (Illumina, San Diego, CA, USA). The detailed workflow for the 16S rRNA sequencing data analysis is shown in the Supplementary Materials.

### 2.10. Bile Acid Analysis

The bile acid (BA) analysis was also conducted by TinyGen Biotechnology (Shanghai) Co., Ltd. After freeze-dried feces were ground and added with ethanol, they were centrifuged after ultrasound. The supernatant was removed into the collection tube, and BA was extracted with isopropanol. Then the supernatant was combined and analyzed by LC-MS after filtration.

### 2.11. Western Blot Assay

Total protein content was extracted from mouse colon tissue or Caco-2 cells, followed by quantification using the BCA assay. The cell lysate was heated at 100 °C with 1× loading buffer for 10 min. Protein separation was achieved using a 10% SDS-PAGE gel and then transferred onto PVDF membrane. Then, primary antibodies (1:1000) were incubated overnight at 4 °C, and secondary antibodies (1:5000) were incubated overnight at room temperature after blocking with 5% BSA. The protein bands were visualized by chemiluminescent detection system with ECL substrate (Yeasen, Shanghai, China). The gray values of the bands were analyzed using ImageJ software (v1.8.0.345).

### 2.12. Immunohistochemical Staining

The paraffin-embedded colon tissue slices were blocked with goat serum for 1 h at room temperature, followed by incubation with antibodies. The secondary antibody was diluted at a concentration of 1:400 and applied to the slide at room temperature for 1 h. Finally, the samples were observed under a microscope.

### 2.13. Alcian Blue Staining

The tissue section was sliced into 5 μm thick sections and photographed under a microscope after dewaxing, acidification, nuclear re-staining, dehydration, soaking and sealing.

### 2.14. Periodic Acid-Schiff (PAS) Staining

The tissue section was sliced into 5 μm thick sections and photographed under a root microscope after dewaxing, periodic acid oxidation, Schiff reagent treatment, dehydration, and sealing with transparent and neutral gum.

### 2.15. Immunofluorescence

The Caco-2 cells were fixed with 4% paraformaldehyde for 15 min and permeabilized with 0.1% Triton-X 100 for 10 min. The cells were then incubated with Occludin antibody overnight, followed by staining with Alexa Fluor 488 (Molecular Probes, Waltham, MA, USA)-conjugated secondary antibody and DAPI.

### 2.16. Transwell Assays

Caco-2 cells and IEC6 cells at a density of 5 × 10^4^/mL cells per insert were seeded. Caco-2 cells were cultivated for 21–30 days, and IEC6 cells were cultivated for 7 days. The treatment group used a solution containing 1 μM of the drug and 1 μg/mL LPS prepared in cell culture medium, incubating the cells for 24 h. The model group was stimulated with only 1 μg/mL LPS. After 24 h, 100 μL FITC-dextran at a concentration of 1 μg/mL was added to the chamber. After 4 h, 100 μL of culture medium in the lower chamber was collected and added to the 96-well microplate, and the fluorescence value was measured to calculate the concentration of fluorescence leakage.

### 2.17. Immunofluorescence (IF)

Following drug treatment of each cell group, immunofluorescence staining was performed using the following steps, with cells analyzed via confocal microscopy: (1) Fixation: remove medium, add 100 μL 4% paraformaldehyde to fix cells, incubate at room temperature for 15 min; (2) Membrane permeabilization: discard fixative, gently wash cells twice with PBS. Add 100 μL PBS containing 3% Triton X-100 for membrane permeabilization, incubate at room temperature for 15 min; (3) Blocking: discard Triton X-100, gently wash cells twice with PBS. Add 100 μL PBS containing 10% goat serum for blocking, incubate at room temperature for 30 min; (4) Primary antibody incubation: discard the blocking solution. Add 50 μL of primary antibody solution to each well and incubate overnight at 4 °C. (5) Secondary antibody incubation: discard the primary antibody. Gently wash the cells twice with PBS. Add 50 μL of fluorescently labeled secondary antibody to each well and incubate at room temperature for 1 h to specifically recognize primary antibody binding sites. (6) DAPI staining and coverslipping: 10 min before the end of secondary antibody incubation, add 1 μg/mL DAPI staining solution to each well to label cell nuclei and coverslip. Analyze cells using a confocal microscope.

### 2.18. Data Availability

Raw sequencing data for all 46 samples in the present study are publicly available at the National Center for Biotechnology Information (NCBl) database with study accession number PRJNA1197721.

### 2.19. Statistics

Data analysis was conducted using GraphPad Prism 8.3.0 software. The experimental results were presented as “mean ± SD”. Statistical differences were assessed by one-way analysis of variance (ANOVE). *p* < 0.05 was considered statistically significant.

## 3. Results

### 3.1. Bile Acid Metabolism Is Implicated in UC and T3K Alleviates DSS-Induced Colitis

**Figure 1:**
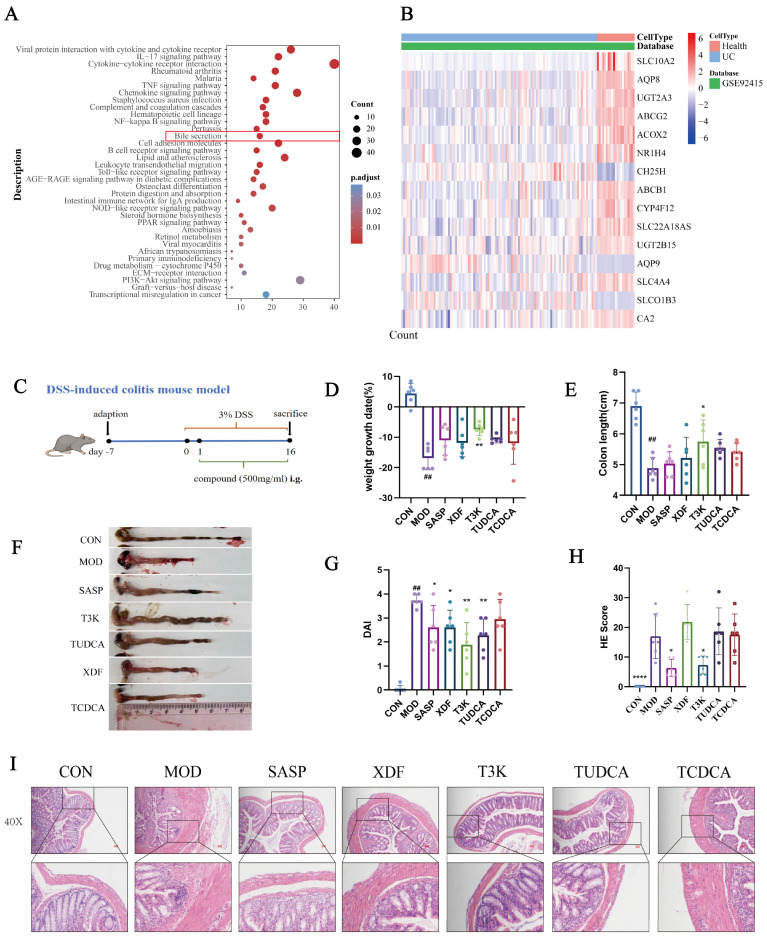
BAs play important roles in UC and bile derivative T3K had therapeutic effect on UC mice. (**A**) Public databases show significant differences in intestinal bacteria and bile acids between UC patients and healthy individuals. KEGG analysis of the public transcriptional UC cohort (UC patients and control individuals). (**B**) Significantly altered expression of various BA-associated transcripts in UC patients compared with normal individuals. (**C**) Schematic outline of the experimental design. (**D**) Changes in body weight in different groups. (**E**) Colon length in different groups. (F) Representative images of the colon. (**G**) DAI in different groups. (**H**) Colonic HE scores of different groups. (**I**) Histopathological change in different groups. (*n* = 6) (^##^, *p* < 0.01, vs. CON; *, *p* < 0.05, **, *p* < 0.01, vs. MOD; ****, *p* < 0.0001, vs. MOD).

To investigate the role of BAs in colitis, we reanalyzed the publicly available patient dataset (GSE92415) from the GEO database [25,26]. Pathway analysis revealed that, in addition to inflammation-related pathways, bile secretion was significantly linked to IBD development, as indicated by KEGG analysis (Figure 1A). Several genes involved in BA metabolism were significantly altered in UC patients (Figure 1B), underscoring the crucial role of BAs in UC pathology.

The structures of TUDCA, TCDCA, and T3K, with the biomimetic synthetic routes for 3K and T3K, are detailed in Supplementary Figure S1. A DSS-induced UC model was generated as previously described (Figure 1C) [27]. Compared to the model group, drained bear bile powder (XDF) and TUDCA significantly improved the disease activity index (DAI) in DSS-induced colitis mice. T3K treatment further alleviated weight loss, reduced DAI, and increased colon length. Colonic tissue from DSS model mice exhibited severe histological damage: colonic architecture was significantly disrupted, characterized by extensive loss or complete disappearance of crypt structures and widespread epithelial cell sloughing. The lamina propria and submucosa showed massive infiltration of inflammatory cells (predominantly neutrophils and lymphocytes), forming distinct inflammatory foci accompanied by severe mucosal edema and hyperemia. Histological analysis was conducted based on the criteria established by Dieleman LA et al. [26]. The DSS model group received the highest pathological damage score, indicating severe acute colitis. In contrast, the T3K (500 mg/kg) treatment group showed significantly improved colonic histopathology. The degree of inflammatory cell infiltration was markedly reduced, with a noticeable decrease in inflammatory foci and edema. Damage to crypt structures was mitigated, with partial crypt preservation observed and improved maintenance of epithelial layer integrity. T3K showed greater efficacy than SASP, XDF, TUDCA, and TCDCA (Figure 1D–I).

### 3.2. T3K Restores Intestinal Barrier Integrity and Reduces Epithelial Permeability

**Figure 2:**
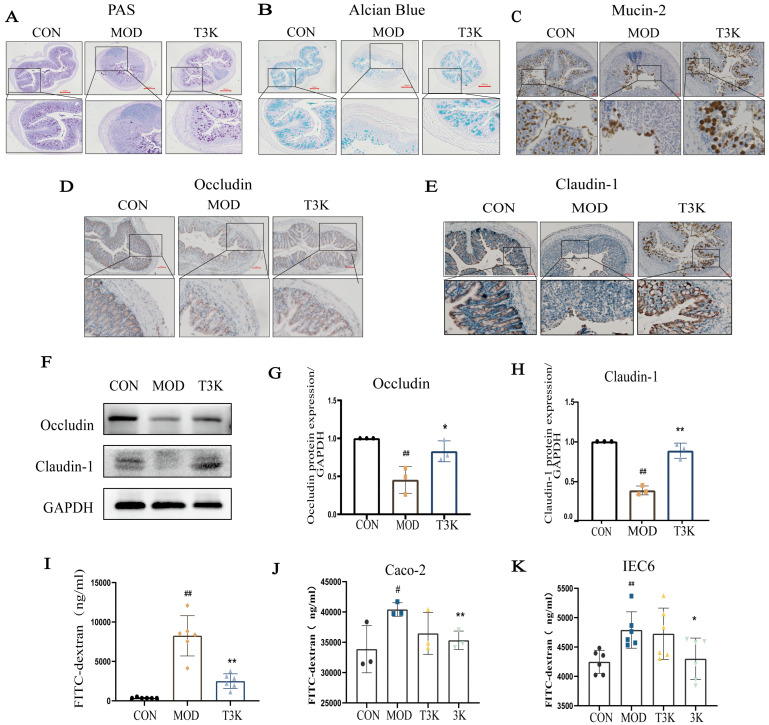
T3K restored intestinal barrier in DSS-induced colitis. (**A**) Representative PAS staining of the colonic sections (*n* = 3). (×40) Scale bar = 100 µm. (**B**) Representative Alcian Blue staining (×40) of the colonic sections (*n* = 3). Scale bar = 100 µm. (**C**–**E**) Representative images of the immunohistochemistry of Muc2, Occludin and Claudin-1 of the colonic sections. Scale bar = 100 µm (*n* = 3). (**F**) Representative Western blot of Occludin and Claudin-1 (*n* = 3). (**G**,**H**) Relative expression levels of Occludin and Claudin-1, data are presented as the mean ± SD (*n* = 3). (**I**) Epithelial permeability of FITC-dextran in colitis mice (*n* = 6). (**J**) Epithelial permeability of FITC-dextran in Caco-2 cells (*n* = 3). (**K**) Epithelial permeability of FITC-dextran in IEC6 cells (*n* = 3). (^#^, *p* < 0.05, vs. CON; ^##^, *p* < 0.01, vs. CON; *, *p* < 0.05, **, *p* < 0.01, vs. MOD).

The mucosal barrier, comprising goblet cells and their secreted mucins, is essential for preventing direct contact between intestinal bacteria and colonic epithelial cells. PAS and Alcian blue staining were used to measure neutral and acidic polysaccharide mucins in the intestinal mucosa, indirectly reflecting the barrier’s function. T3K treatment significantly increased goblet cell numbers and expression of mucin (Figure 2A,B). Mucin2 (Muc2), produced by goblet cells, is a key structural component of the mucosal barrier [28]. Immunohistochemical analysis showed that Muc2 levels were significantly reduced in the model group but were restored by T3K treatment (Figure 2C). Tight junction (TJ) proteins, including Occludin and Claudin-1, were analyzed to further assess epithelial integrity. Occludin and Claudin-1 levels were markedly decreased in the model group and significantly increased with T3K treatment (Figure 2D–H).

Increased intestinal permeability is a key feature in both UC patients and animal models. Disruptions in mucins and TJ proteins contribute to this heightened permeability. FITC-dextran levels, used as a permeability marker, were significantly higher in the model group compared to controls but were reduced following T3K treatment, indicating improved barrier function (Figure 2I). To confirm these effects in vitro, a transwell monolayer model with Caco-2 and IEC6 cells was used to evaluate T3K’s protective effects on the intestinal barrier. Notably, T3K, a tauro-conjugated bile salt, has enhanced solubility. In a biological context, T3K undergoes deconjugation facilitated by bile salt hydrolase (BSH), an enzyme abundant in the gut microbiome. This process is akin to that of TCDCA and TCA, leading to the release of 3K, which then enters systemic circulation to participate in various metabolic pathways. As T3K remains inactive in vitro, all validation experiments utilized 3K. FITC-dextran leakage significantly increased in the model group but was effectively reduced by 3K (Figure 2J–K). These results confirm that T3K effectively restores intestinal barrier integrity.

### 3.3. T3K Rectifies Gut Microbial Dysbiosis in Colitis Mice

**Figure 3:**
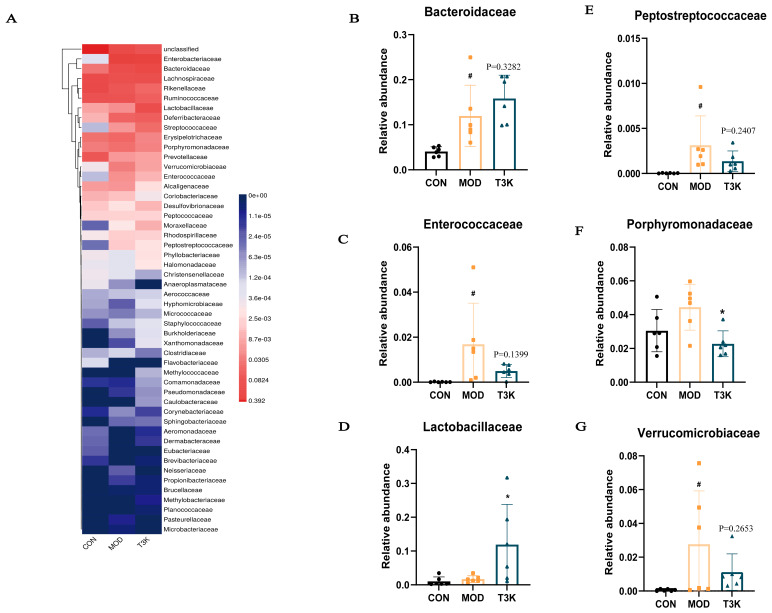
T3K relieved the microbiota dysbiosis in the colitis mice. (**A**) Structure of community analysis at the family level (*n* = 6). (**B**) The abundance of Bacteroidaceae in different groups. (**C**) The abundance of *Enterococccaceae* in different groups. (**D**) The abundance of *Lactobacillaceae* in different groups. (**E**) The abundance of *Peptostreptococccaceae* in different groups. (**F**) The abundance of *Porphyromonadaceae* in different groups. (**G**) The abundance of *Verrucomicrobiaceae* in different groups. (^#^, *p* < 0.05, vs. CON; *, *p* < 0.05).

Dysbiosis of the intestinal microbiota is a prominent pathological characteristic of UC, marked by significant changes in gut microbiota composition. Heatmap analysis showed T3K improved gut microbiota disorders compared with the DSS model group (Figure 3A). LDA Effect Size analysis (LEfSe), indicating the phylogenetic distribution, was used to determine significant differences in relative abundance of mice intestinal flora with DSS colitis (presented as red column) compared with control mice (blue) and T3K treatment (green), and T3K improved beneficial microbiota (Supplementary Figure S2). Relative abundance analysis further revealed that T3K significantly increased beneficial microbiota, such as *Lactobacillaceae* and *Bacteroidaceae*, while decreasing pathogenic microorganisms like *Enterococcus*, *Peptostreptococcaceae* and *Porphyromonadaceae* (Figure 3B–G). In summary, T3K effectively alleviated dysbiosis of the intestinal microbiota and mitigated DSS-induced colitis in mice.

### 3.4. T3K Normalizes Bile Acid Metabolism and Increases Hydrophilic Bile Acids

**Figure 4:**
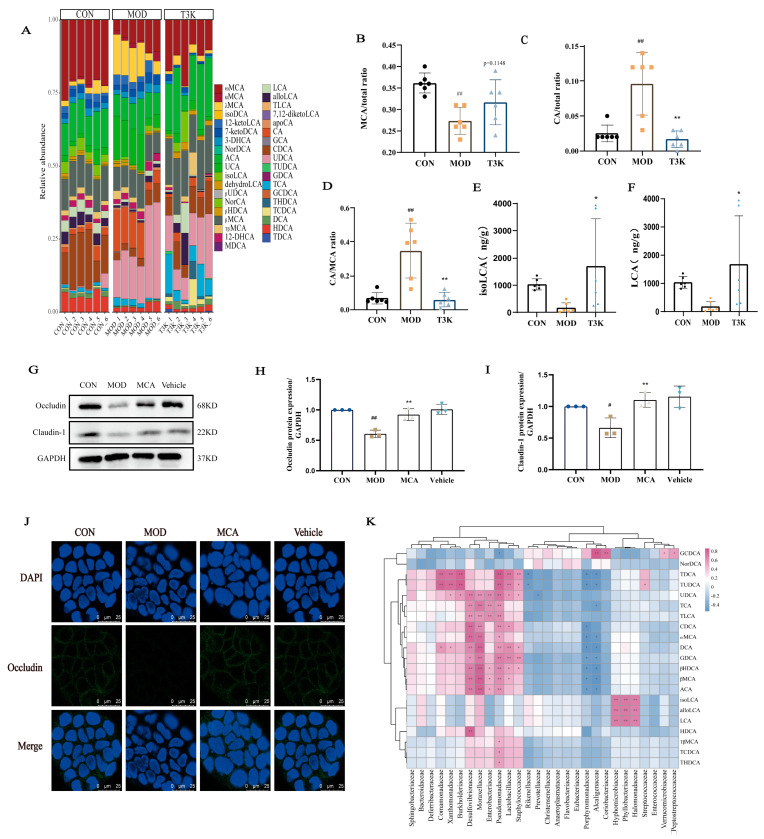
T3K improved BA metabolism in the colitis mice. (**A**) Composition of bile acid pool in feces (*n* = 6). (**B**) MCA ratio in colitis mice bile acid pool (*n* = 6). (**C**) CA ratio in colitis mice bile acid pool (*n* = 6). (**D**) CA/MCA ratio in colitis mice bile acid pool (*n* = 6). (**E**,**F**) The level of isoLCA and LCA between different groups. (**G**) Representative Western blot of Occludin and Claudin-1 in Caco-2 cells treated with DSS and MCA. (**H**,**I**) Relative expression levels of Occludin and Claudin-1, data are presented as the mean ± SD (*n* = 3). (**J**) Representative immunofluorescence images showing expression of Occludin in Caco-2 cells treated with DSS and MCA. Scale bar = 25 µm. (**K**) Pearson correlation analysis shows the correlation between fecal BAs and gut microbiota. The spot with an asterisk in pink refers to the significant positive correlation, and blue indicates negative correlation (*n* = 6) (^#^, *p* < 0.05, vs. CON; ^##^, *p* < 0.01, vs. CON; *, *p* < 0.05, **, *p* < 0.01, vs. MOD).

BAs are key microbial metabolites that stabilize the intestinal immune microenvironment through microbial metabolism and transformation [29,30]. Dysregulation of BA metabolism has been linked to the onset and progression of IBD, highlighting the essential role of BAs in maintaining intestinal homeostasis. Results of bile abundance revealed that the BA profile in the T3K group closely resembled that of the control group, in contrast to the model group (Figure 4A). This indicates that T3K effectively improves the composition of BAs in the intestinal tract, mitigating disturbances in BA metabolism. Hydrophilic BAs, such as muricholic acid (MCA), help stabilize lipid membranes, while hydrophobic BAs, such as cholic acid (CA), can disrupt liposomes and increase barrier permeability by permitting the translocation of bacteria and antigens across the intestinal barrier, potentially leading to inflammation [31,32]. The heat map results demonstrated that T3K significantly elevated hydrophilic BAs like MCA, decreased the proportion of hydrophobic BAs like CA, and improved the CA/MCA ratio (Figure 4B–D). T3K also increased the level of secondary bile acid such as lithocholic acid (LCA) and Isolithocholic Acid (isoLCA) (Figure 4E,F).

CA has been shown to compromise intestinal integrity by affecting TJs [32], and then the effects of MCA on TJs were investigated in this study instead. Western blot revealed that MCA significantly increased the expression of TJ proteins Occludin and Claudin-1 in Caco-2 cells co-treated with MCA and DSS (Figure 4G–I). Immunofluorescence analysis further confirmed that MCA significantly upregulated Occludin expression (Figure 4J). These findings suggest that T3K may enhance intestinal barrier stability by increasing MCA levels and decreasing CA levels.

Additionally, based on 16S rRNA sequencing (microbiome composition) and targeted bile acid LC-MS/MS quantitative data, Pearson correlation analysis was performed between fecal bile acids and the gut microbiome. It revealed significant associations between changes in BA profiles and variations in gut microbial composition (Figure 4K). For example, beneficial bacteria such as *Lactobacillaceae* positively correlated with β-MCA and other BAs, while *Porphyromonadaceae* showed a negative correlation with β-MCA and ω-MCA. *Porphyromonadaceae* is recognized as a major pathogenic bacterium linked to various metabolic disorders, including IBD [33]. These results indicate that the hydrophilicity of MCA may exceed that of CA, allowing T3K to enhance the proportion of hydrophilic BAs and support the restoration of the intestinal barrier.

### 3.5. T3K Loses Efficacy in Alleviating Symptoms of DSS-Induced Colitis in PGF-Mice

**Figure 5:**
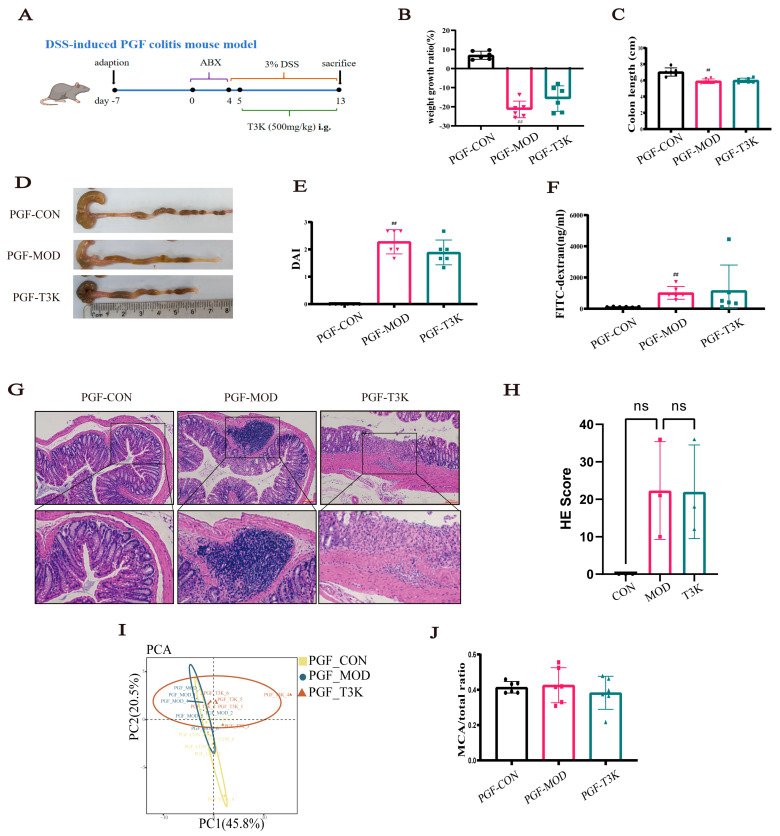
T3K loss its effect on UC without the gut microbiota. (**A**) Schematic outline of PGF UC model. (**B**) Changes in body weight in different groups (*n* = 6). (**C**) Colon length in different groups (*n* = 6). (**D**) Representative images of the colon. (**E**) DAI in different groups (*n* = 6). (**F**) Epithelial permeability of FITC-dextran in colitis mice treated by ABX (*n* = 6). (**G**) Representative H&E staining (×100) of the colonic sections (*n* = 3). Scale bar = 100 µm. (**H**) Colonic HE scores of different groups (*n* = 3) (**I**) Principal component analysis plots of BA in different groups (*n* = 6). (**J**) MCA ratio in PGF-colitis mice bile acid pool. (*n* = 6) (^##^, *p* < 0.01, vs. CON; ns, not significant).

To determine whether T3K can restore disturbances in BA metabolism by modulating intestinal microbes, we utilized PGF mice to simulate a germ-free environment (Figure 5A). Our results showed that, although colitis was successfully induced in PGF mice, T3K had no significant impact on UC parameters such as weight loss, DAI scores, and colonic contracture after intestinal bacteria were cleared with an antibiotic cocktail (Figure 5B–E). The dependence of T3K’s effectiveness on gut microbiota was further verified by pathological analyses of colon tissues from pseudo-germ-free (PGF) mice (Figure 5G,H). The colonic mucosal morphology of the PGF-control group (PGF-CON) remained normal, with intact crypts and no inflammatory infiltration. The PGF-model group (PGF-MOD) displayed the typical pathological characteristics of DSS-induced colitis, such as extensive epithelial detachment, significant tissue edema (increased mucosal and submucosal thickness due to fluid accumulation), severe crypt destruction (widespread loss of crypt structure, reduced crypt depth, and irregular arrangement), and dense inflammatory cell infiltration (neutrophils and macrophages) in the damaged areas. In the PGF-MOD group, focal ulcerations with fibrous exudate coverage were also noted. In contrast, the PGF-T3K group showed no significant improvement in these pathological indicators compared to the PGF-MOD group. The colonic mucosa of PGF-T3K mice still exhibited obvious crypt damage, epithelial discontinuity, and substantial inflammatory cell infiltration. Quantitative histological scoring (based on inflammation severity, crypt destruction, edema degree, and ulceration area) revealed no statistically significant difference between the PGF-T3K and PGF-MOD groups (Figure 5H). These results indicated that in the absence of gut microbiota, T3K failed to alleviate colonic pathological damage, further supporting that the therapeutic effect of T3K on colitis is mediated by regulating intestinal microbiota. Assessment of intestinal permeability in PGF mice revealed that T3K lost its efficacy under these conditions (Figure 5F). These findings suggest that T3K may exert its pharmacological effects through the modulation of intestinal microorganisms. Further analysis of BAs in the fecal samples of PGF mice revealed no significant differences in BA pools among the groups (Figure 5I), indicating no notable dysregulation of BA metabolism. Moreover, the ratio of hydrophilic MCA did not differ significantly between the PGF-T3K and PGF-MOD groups, suggesting that T3K did not affect the proportion of hydrophilic BAs in PGF mice (Figure 5J). In summary, the absence of intestinal microbiota in these mice negates the effects of T3K on DSS-induced UC, thereby diminishing its impact on microbial composition and BA metabolism.

### 3.6. T3K Attenuates DSS-Induced Colitis Through Intestinal Microbiota Modulation

**Figure 6:**
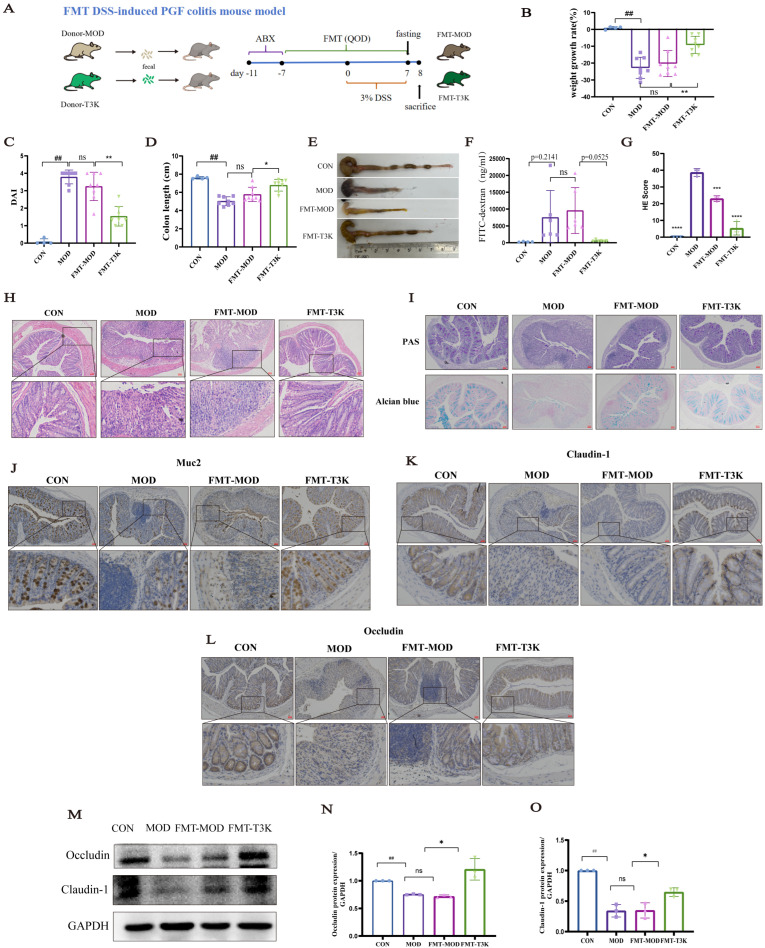
T3K attenuated DSS-induced colitis relying on the gut microbiota. (**A**) Schematic outline of FMT in the colitis mice. (**B**) Changes in body weight in different groups (*n* = 8). (**C**) DAI in different groups (*n* = 8). (**D**) Colon length in different groups (*n* = 8). (**E**) Representative images of the colon. (**F**) Epithelial permeability of FITC-dextran in FMT colitis mice (*n* = 6). (**G**) Colonic HE scores of different groups (*n* = 3) (**H**) Representative H&E staining (×100) of the colonic sections of FMT colitis mice (*n* = 3). (**I**) Representative PAS staining and Alcian Blue staining (×100) of the colonic sections (*n* = 3). Scale bar = 100 µm. (**J**–**L**) Representative images of the immunohistochemistry of Muc2, Occludin and Claudin-1 of the colonic sections (*n* = 3). Scale bar = 100 µm. (**M**) Representative Western blot of Occludin and Claudin-1 (*n* = 3). (**N**,**O**) Relative expression levels of Occludin and Claudin-1, data are presented as the mean ± SD (*n* = 3). (^##^, *p* < 0.01, vs. CON; *, *p* < 0.05, **, *p* < 0.01,***, *p* < 0.001,****, *p* < 0.0001 vs. FMT-MOD).

To investigate the specific role of intestinal microbiota in the therapeutic effects of T3K, we established the FMT DSS-induced PGF colitis mouse model. Fecal samples from untreated and T3K-treated mice were transplanted into PGF mice, as illustrated in Figure 6A. The FMT-MOD group was successfully established, showing no significant differences compared to the MOD group. In contrast, the FMT-T3K group demonstrated significant improvements in weight, reductions in DAI scores, enhanced colon contracture, and increased colon length, effectively alleviating colon inflammation (Figure 6B–E). Serum levels of FITC-dextran were significantly elevated in the MOD and FMT-MOD groups but markedly reduced in the FMT-T3K group, indicating decreased intestinal permeability (Figure 6F). HE staining revealed that FMT-T3K reduced colonic edema and mucosal damage, restoring crypt architecture (Figure 6G,H). The FMT-MOD group, which received microbiota from DSS-induced donors, developed colitis pathology similar to the conventional MOD group, featuring mucosal ulceration, crypt abscesses, and moderate to severe inflammatory cell infiltration. Conversely, the FMT-T3K group, transplanted with microbiota from T3K-treated donors, demonstrated remarkable mucosal healing. The epithelium was largely continuous, crypts were elongated and well-structured with rare abscesses, and inflammation was markedly reduced to mild, patchy foci within the lamina propria. Periodic Acid-Schiff (PAS) and Alcian blue staining results showed a significant decrease in mucin content and goblet cell counts in the FMT-MOD group. In contrast, the FMT-T3K group exhibited a substantial increase in mucin expression, effectively preserving goblet cell populations in the colonic mucosa (Figure 6I). Immunohistochemical analysis further revealed a significant increase in Muc2 abundance in the FMT-T3K group (Figure 6J). Moreover, the expression of TJ proteins Occludin and Claudin-1 was elevated in the FMT-T3K group (Figure 6K–M). Analysis of Occludin and Claudin-1 levels in the colon showed that Occludin and Claudin-1 levels were significantly reduced in the model group, while their protein content increased in the T3K group (Figure 6N,O).

In summary, these results indicate that T3K enhances intestinal barrier integrity and exerts anti-UC effects through a gut microbiota-dependent mechanism.

### 3.7. T3K Restores Gut Flora and BA Metabolism via Fecal Microbiota Transplantation

**Figure 7:**
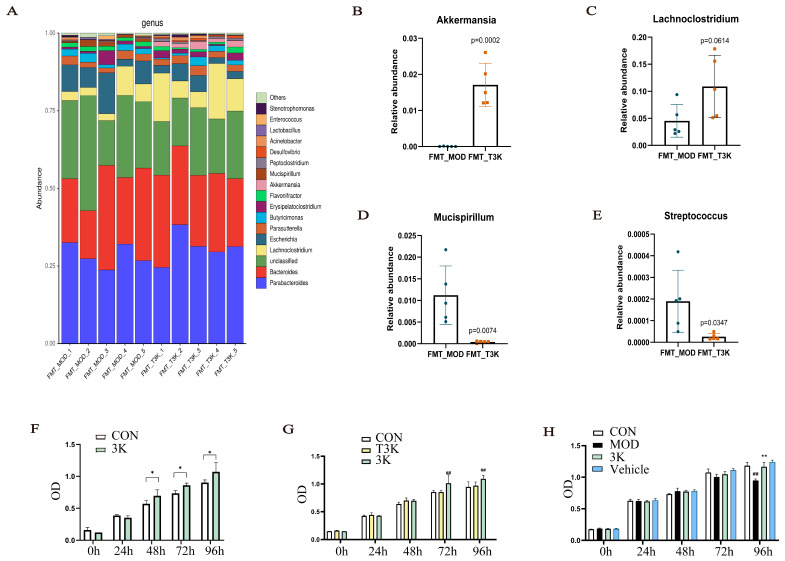
T3K regulated the composition of intestinal microbiota by FMT. (**A**) Structure of community analysis at the genus level (*n* = 5). (**B**) The abundance of *Akkermansia* in different groups. (**C**) The abundance of Bacteroidaceae in different groups. (**D**) The abundance of Bacteroidaceae in different groups. (**E**) The abundance of Bacteroidaceae in different groups. (**F**) The proliferative effect of 3K on *Akkermansia muciniphila* (*n* = 5). (**G**) The differential proliferative effect on *Akkermansia muciniphila* between 3K and T3K (*n* = 5). (**H**) The rescue effect of 3K on the proliferation of *Akkermansia muciniphila* with 3%DSS in vitro. (^##^, *p* < 0.01, vs. CON; *, *p* < 0.05, **, *p* < 0.01, vs. FMT-MOD).

Analysis indicated that *Akkermansia muciniphila* was the dominant species in this group (Figure 7A). The abundance of *Akkermansia muciniphila* significantly increased in the FMT-T3K group (Figure 7B). There was also a significant increase in *Lachnoclostridium* and a marked decrease in *Mucispirillum* in the FMT-T3K group at the genus level (Figure 7C,D). *Streptococcus*, as one of the potentially detrimental bacteria in IBD, was also inhibited in the FMT-T3K group (Figure 7E). *Lachnoclostridium* has been reported to be associated with immunoregulatory genes [34], and *Akkermansia muciniphila* is positively associated with IBD [35]. The proliferative effect of T3K on *Akkermansia muciniphila* was further confirmed through anaerobic culturing of this bacterium, treated with 3K and T3K (1μM) for 24 to 96 h in vitro. Results demonstrated that 3K significantly enhanced the growth of *Akkermansia muciniphila* between 48 and 96 h (Figure 7F,G). In vitro exposure to 3% DSS led to a significant decline in *Akkermansia muciniphila* abundance and impaired its proliferative capacity; however, 3K effectively restored this proliferation (Figure 7H). These results suggest that T3K effectively modulates the gut microbiota in mice, promoting beneficial species especially including *Akkermansia muciniphila* while reducing harmful ones.

**Figure 8:**
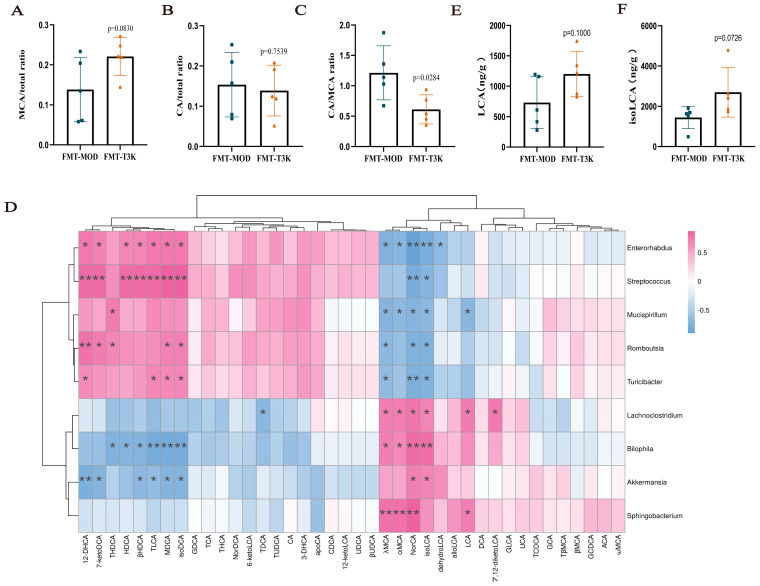
T3K regulated the bile acid dysmetabolism by FMT. (**A**–**C**) The effect of T3K on MCA, CA and CA/MCA (*n* = 5) between FMT-MOD and FMT-T3K. (**D**) Pearson correlation analysis shows the correlation between differential fecal BAs and gut microbiota. (**E**,**F**) The level of LCA and isoLCA between FMT-MOD and FMT-T3K (*n* = 5). (*, *p* < 0.05, **, *p* < 0.01, vs. MOD).

We further analyzed the BA and its relationship with intestinal flora between the FMT-MOD and FMT-T3K groups (Figure 8). Statistically, the FMT-T3K group showed an increased proportion of MCA, a significantly decreased proportion of CA/MCA and, finally, a favorable regulation of hydrophilic BA proportions compared to the FMT-MOD group (Figure 8A–C). Correlation analysis between distinct intestinal bacteria and specific BAs revealed MCA is positively correlated with beneficial bacteria such as *Lachnoclostridium* and negatively correlated with *Mucispirillum* and other bacteria (Figure 8D). The level of LCA and isoLCA was also increased in the FMT-T3K group (Figure 8E,F). In summary, results from both PGF and FMT experiments indicate that T3K alleviates DSS-induced colitis by enhancing intestinal flora and modulating BA metabolism disorders, thereby repairing the intestinal mucosal barrier.

## 4. Discussion

UC is a chronic inflammatory disease characterized by abdominal pain, diarrhea, bloody stools, and pathological mucosal damage, including ulcers in the intestine [36]. The etiology of UC is multifactorial, including immune dysregulation, disturbances in intestinal microbiota, and genetic predisposition [37]. We analyzed differential gene expression between UC patients and healthy individuals using the public NCBI database, revealing significant changes in genes related to BA metabolism (Figure 1A,B). An imbalance in BA metabolism is closely associated with the onset of UC.

Clinical trials and preclinical studies have explored the therapeutic potential of bear bile powder (XDF) and various BA derivatives, including TUDCA, for treating UC. We successfully synthesized the BA derivatives T3K and 3K through a series of enzymatic and chemical reactions starting from chenodeoxycholic acid (CDCA), as shown in Supplementary Figure S1. Notably, T3K, a tauro-conjugated bile salt, has enhanced solubility. In a biological context, T3K undergoes deconjugation facilitated by bile salt hydrolase (BSH), an enzyme abundant in the gut microbiome. This process is akin to that of TCDCA and TCA, leading to the release of 3K, which then enters systemic circulation to participate in various metabolic pathways. As T3K remains inactive in vitro, all validation experiments utilized 3K.

The DSS-induced colitis mouse model is commonly employed to simulate UC symptoms, such as weight loss, diarrhea, and bloody stools [38,39,40]. In our studies, T3K demonstrated greater efficacy than XDF, TCDCA, and TUDCA, significantly alleviating UC symptoms, including weight loss, colon contracture, and increased DAI scores (Figure 1D–I). HE staining results further confirmed that T3K effectively improved colonic tissue structure and reduced inflammatory infiltration. Overall, this study highlights the potential of T3K to relieve symptoms of colitis (Figure 1).

The invasion of pathogenic microorganisms and endotoxins through a compromised intestinal barrier triggers inflammation, highlighting the importance of restoring this barrier for the effective management of UC. The intestinal barrier consists of a mucus layer, commensal bacteria, epithelial cells, and immune cells in the lamina propria [41]. Goblet cells in the colonic epithelium secrete mucus glycoproteins that create a physical barrier between the gut microbiome and intestinal epithelial cells, providing essential protection [42]. Studies have shown that mice lacking Mucin 2 develop spontaneous colitis and may be more prone to colorectal cancer. These findings emphasize the critical role of mucin in defending against pathogens and maintaining intestinal barrier integrity [43,44]. Our results reveal that T3K significantly elevates total mucin levels and Mucin 2 expression, thereby enhancing the integrity of the intestinal mucus barrier in mice (Figure 2A–C). Mechanistically, Muc2 is essential for the formation of a gel-like mucus layer that traps beneficial bacteria and keeps pathogens out. Previous research demonstrating that Muc2 overexpression decreases intestinal colonization by pro-inflammatory microbes supports the idea that T3K-induced upregulation of Muc2 may directly inhibit the adherence of pathogenic bacteria, e.g., Enterococcaceae (Figure 3D).

Adjacent intestinal epithelial cells form a cohesive network through various proteins, including TJs, adherens junctions, desmosomes, and gap junctions, extending from the apical surface to the basement membrane. TJs are essential for maintaining cell–cell adhesion and barrier function [45], primarily comprising Occludin, Claudins, and Zonula Occludens-1 (ZO-1) [46]. Our findings demonstrate that T3K effectively promotes the repair of the colonic epithelium and upregulates the expression of Occludin and Claudin-1. We hypothesize that T3K primarily enhances the expression of transmembrane TJ proteins, particularly Occludin and Claudin-1 and then reduces intestinal permeability (Figure 2D–K).

Commensal microbes in the gut not only serve as mechanical barriers against pathogens but also play a crucial role in maintaining gut homeostasis. The microbial barrier is essential for the onset and progression of UC. Metagenomic sequencing of fecal samples from patients with IBD has shown a significant decrease in the abundance, functional diversity, and stability of intestinal bacteria compared to healthy individuals [47]. Additionally, these studies reveal marked changes in the composition of microbial communities, with increased pathogenic bacteria and decreased beneficial bacteria, suggesting a potential link to the development or exacerbation of intestinal inflammation [47,48]. Research on the stool microbiota of IBD patients indicates a reduction in *Bacteroidetes* and *Firmicutes*, coupled with an increase in *Proteobacteria* and *Actinobacteria* [5,49,50,51]. Medications and probiotics can effectively modulate the gut microbiome, restore homeostasis, and alleviate intestinal inflammation in IBD patients [52]. Our results demonstrate that T3K alleviates colitis-induced dysbiosis by promoting the proliferation of beneficial bacteria, such as *Bacteroidaceae* and *Lactobacillaceae* and, especially, inhibiting harmful bacteria including *Enterococcaceae*, *Peptostreptococcaceae* and *Porphyromonadaceae* (Figure 3). Notably, *Porphyromonadaceae* are linked to increased intestinal permeability and TNF-αsecretion [33], whereas *Lactobacillaceae* are known to produce short-chain fatty acids (SCFAs) like butyrate, which increase TJ protein expression and inhibit pro-inflammatory cytokine production [53]. In line with earlier findings that microbial dysbiosis correction is a crucial mechanism for anti-UC agents, T3K-induced alterations in these bacterial families (Figure 3C,G) may synergistically improve gut barrier function and reduce inflammation. Overall, these results suggest that T3K enhances the functionality of the mucus, mechanical and microbial barriers of the intestine.

Specific metabolites produced by intestinal microbiota, such as BAs, short-chain fatty acids and tryptophan metabolites, are closely associated with the development of IBD [12,13]. Among various metabolic factors, abnormal BA metabolism is significantly linked to the onset and progression of UC [54,55,56]. BAs mainly comprise primary BAs like CA and CDCA, as well as secondary BAs such as deoxycholic acid (DCA) and lithocholic acid (LCA), which are produced through the metabolism of intestinal bacteria. Clinical studies show that approximately 10% of IBD patients also have primary sclerosing cholangitis (PSC), and around 80% of PSC patients develop IBD, particularly UC [12]. But the effect of BA on gut homeostasis remains poorly characterized and controversial. Primary and secondary BAs, particularly those with high hydrophobicity such as CA, have been reported to exacerbate colitis [32,57]. But in some reports, secondary BAs promoted intestinal stem cell proliferation and tissue repair in UC [58]. A specific combination of primary or secondary BAs with long-term feeding can also ameliorate UC [30]. In our study, we identified over 40 different BAs in mouse feces and demonstrated that T3K effectively ameliorates BA metabolism disorders. T3K treatment resulted in increased levels of hydrophilic BAs, particularly MCA (Figure 4). Hydrophilic BAs help stabilize the intestinal lipid membrane, whereas hydrophobic BAs may increase intestinal permeability, facilitating the translocation of bacteria and antigens across the barrier, which can lead to inflammation [31,32]. We deduced that T3K may elevate hydrophilic BAs like MCA, decrease the proportion of hydrophobic BAs like CA and finally improve the CA/MCA ratio (Figure 4B–D). Secondary bile acids such as LCA and isoLCA, as metabolites of gut microbiota, are traditionally known for their roles in lipid digestion and antibacterial defense, and are now considered key signaling molecules that regulate metabolism and inflammation [53,59]. The content of LCA and isoLCA in the intestine was significantly reduced compared to the healthy control group and LCA administration significantly alleviates colitis symptoms [53,60]. T3K could also elevate the level of LCA and isoLCA (Figure 4E,F).

We further investigated whether the impact of T3K on BA metabolism and its capacity to restore intestinal barrier function rely on the gut microbiome, using gnotobiotic (PGF) mouse models and FMT. In the PGF mouse model, T3K did not alleviate symptoms of UC or improve BA metabolism (Figure 5). These results indicate that T3K’s effects on BA metabolism and intestinal barrier function are dependent on gut microbiota. Subsequent FMT experiments demonstrated that feces from T3K-treated mice could restore the beneficial effects of T3K in colitis mice, including colon contracture, DAI scores, intestinal permeability, expressions of TJs and then intestinal barrier integrity (Figure 6), correcting microbial dysbiosis (Figure 7) and BA metabolic disorders (Figure 8). Notably, *Akkermansia muciniphila* emerged as the predominant bacterium in the FMT-T3K group, showing a significant increase in its relative abundance (Figure 7A–C). Previous studies have identified *Akkermansia muciniphila* as a probiotic bacterium that strengthens intestinal barrier integrity, regulates immune function, and reduces inflammation. Xinyu Zhang et al. revealed 19 co-upregulated metabolites, with cucurbitacin E and antcin K being identified as key metabolites within anti-inflammatory pathways modulated by *Akkermansia muciniphila* [61]. Herrera-deGuise C et al. reported there was a positive correlation between *Akkermansia muciniphila* abundance and time in remission of UC patients [62]. Stool microbiota of UC patients who achieved clinical remission showed increased Bacteroidetes and decreased Proteobacteria abundances, along with an enrichment of beneficial taxa, including *Bifidobacterium* and *Akkermansia muciniphila* [63]. In vitro experiments demonstrated that 3K significantly promotes the proliferation of *Akkermansia muciniphila* (Figure 7F–H). In addition, multiple *Akkermansia muciniphila* strains have already been investigated. The website Probio-Ichnos provides access to information on currently isolated strains and their in vitro probiotic properties [64] (Supplementary Table S1). Additionally, Xianfeng Guo et al. isolated 40 strains from human and mouse fecal samples. Phylogenetic analysis identified three species-level *A. muciniphila* clades, revealing significant population genomic diversity, functional specificity, geographic distribution, and ecological adaptability within the mucin-loving acidobacteria [65]. Kang FJ et al. isolated the AHG0001 strain from the feces of healthy individuals. Its secreted protein Amuc_1409 activates the Wnt/β-catenin pathway, promotes intestinal stem cell proliferation and differentiation, and enhances mucus layer thickness and barrier integrity [66]. The above research provides direction for our future studies on *A*. *muciniphila*.

In addition to further increasing probiotics, including *Akkermansia* and *Lachnoclostridium*, and reducing harmful bacteria such as *Mucispirillum* and *Streptococcus*, the FMT-T3K group exhibited increased hydrophilic MCA and then decreased the ratio of CA/MCA, thereby enhancing intestinal barrier function (Figure 8A–C). MCA is positively correlated with beneficial bacteria such as *Lachnoclostridium* and negatively correlated with *Mucispirillum* and other bacteria (Figure 8D). It is worth mentioning that LCA and isoLCA not only show similar correlations with MCA, but also that isoLCA shows significant positive correlations with *Akkermansia*. It was reported that human gut bacteria, including *Bacteroides*, *Lachnospira* and *Lactobacillus,* converted LCA into 3-oxoLCA and isoLCA [29]. LCA and isoLCA suppressed Th17 cell differentiation and then modulated gut immune homeostasis during the pathophysiology of inflammatory bowel disease [67,68]. These results suggest that T3K may regulate BA metabolic disorders in UC by modulating intestinal flora, including *Akkermansia muciniphila*, thereby indirectly improving the proportion of BA, including CA/MCA and increasing LCA and isoLCA (Figure 8E,F). Of course, the detailed role of *Akkermansia muciniphila*, LCA and isoLCA in the therapeutic effects of T3K needs further exploration.

## 5. Conclusions

In summary, T3K, a monomeric compound, demonstrates notable anti-UC activity in vivo. Further studies using gnotobiotic (PGF) mouse models and FMT reveal that T3K effectively alleviates intestinal microbial dysbiosis by enhancing beneficial bacterial populations. This modulation of the gut microbiome positively affects BA metabolism, particularly by increasing the levels of MCA, LCA and isoLCA, thereby restoring intestinal barrier function.

## Data Availability

Raw sequencing data for all 46 samples in the present study are publicly available at the National Center for Biotechnology Information (NCBl) database with study accession number PRJNA1197721. And all information has been included in the manuscript.

## Supplementary Materials

The following supporting information can be downloaded at: https://www.mdpi.com/article/10.3390/pharmaceutics18010020/s1, Figure S1. (A) The structure of TUDCA, TCDCA and T3K. (B) The biomimetic synthetic route of 3KDCA (3K) and T3KDCA (T3K). Figure S2. Linear discriminant analysis (LDA) in the family level (DSS-induced mouse colitis model) (*n* = 6). Figure S3. Structure of community analysis at the species level (FMT DSS-induced PGF colitis mouse model) (*n* = 5). Figure S4. (A) Beta diversity heatmap at the genus level. (B) Beta diversity heatmap at the species level. (C) Combined analysis of species-level sample clustering tree and bar chart. (D) Combined analysis of family-level sample clustering tree and bar chart. (E) Simpson index plot. (F) Shannon index plot. Table S1. Functional potential of multiple *Akkermansia muciniphila* strains.

## Abbreviations

BA	Bile acid
DAI	Disease activity index
XDF	Drained bear bile powder
FMT	Fecal microbiota transplantation
FITC	Fluorescein isothiocyanate
GEO	Gene Expression Omnibus
IBD	Inflammatory bowel disease
LCA	Lithocholic acid
PAS	Periodic Acid-Schiff
PGF	Pseudo-germ-free
SASP	Sulfasalazine
TUDCA	Tauroursodeoxycholate
TCDCA	Taurochenodeoxycholate
T3KDCA	Tauro-7α-hydroxy-3-oxo-5β-cholanoate
UC	Ulcerative colitis
