# Supplementary material for: Bile Derivative T3K Ameliorates Colitis by Regulating the Intestinal Microbiota-Bile Acid Axis
Source: Pharmaceutics. 2025 Dec 23;18(1):20. doi: 10.3390/pharmaceutics18010020 (PMC12844640; doi:10.3390/pharmaceutics18010020)
Supplement: Supplementary file 1 [file pharmaceutics-18-00020-s001.zip › pharmaceutics-4009300-supplementary.pdf]

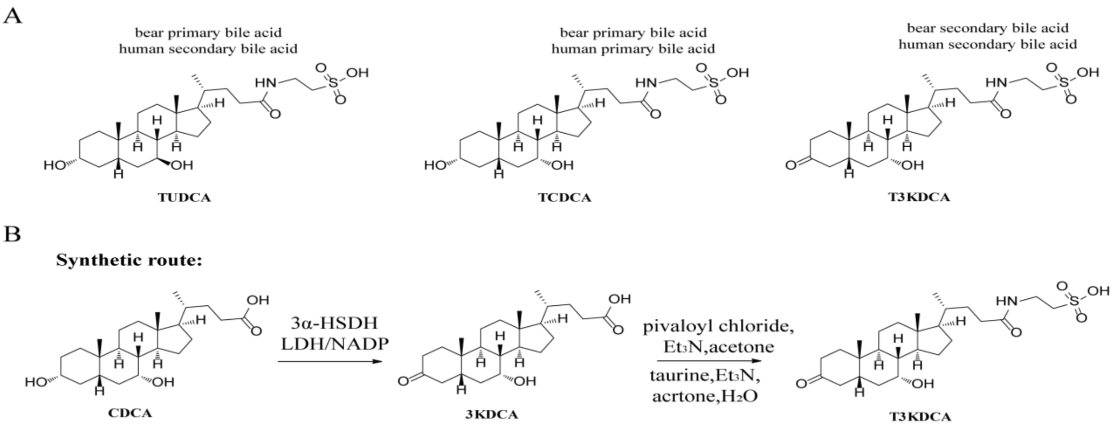

Supplementary Figure S1. (A) The structure of TUDCA, TCDCA and T3K. (B) The biomimetic synthetic route of 3KDCA (3K) and T3KDCA (T3K).

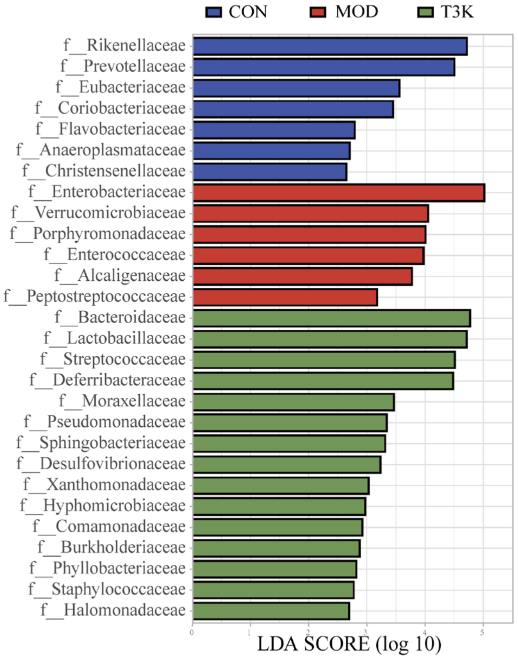

Supplementary Figure S2. Linear discriminant analysis (LDA) in the family level（DSS-induced mouse colitis model） (n=6)

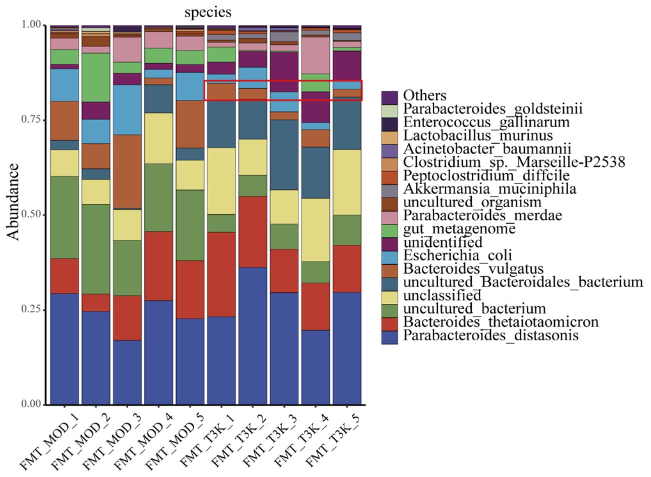

Supplementary Figure S3. Structure of community analysis at the species level（FMT DSS-induced PGF colitis mouse model） (n=5).

The workflow for the 16S rRNA sequencing data analysis

(1) Data Processing Platform and Software： The analysis was primarily conducted using a series of integrated bioinformatics tools. Key software included: Trimmomatic (v0.38) and cutadapt (v1.16) for raw sequence quality control and adapter trimming; FLASH (v1.2.11) for paired-end sequence assembly; USEARCH (v8.1.1861) for OTU clustering and chimera removal; mothur (v1.39.5) for sequence optimization and species taxonomic annotation. Downstream statistical analysis and visualization were performed in the R (v3.6.0) environment using packages such as vegan, ggplot2, and pheapmap.

(2) Sequence Quality Control and Preprocessing: Raw data were first split based on sample-specific barcodes. Strict quality control was then applied: Trimmomatic performed sliding window quality filtering (window size 50 bp, average quality ⩾ Q20) and trimmed reads shorter than 50 bp; cutadapt removed sequencing adapters and primer sequences. FLASH was employed to assemble paired-end reads with parameters set to a minimum overlap length of 10 bp and a maximum mismatch rate of 0.2 in the overlap region. Assembled sequences were further optimized using mothur, which excluded sequences containing ambiguous bases, high homopolymer regions (>8 consecutive bases), and strictly limited sequence lengths to 200 – 485 bp. Singletons (sequences occurring only once) were also removed. (singletons).

(3) OTU Generation and Species Annotation： Traditional OTU clustering was employed instead of the ASV approach. Optimized sequences were clustered using the UPARSE algorithm in USEARCH at a 97% similarity threshold to generate OTU representative sequences. Chimeras were removed by aligning sequences against the gold database (v20110519) using UCHIME. Subsequently, all sequences were mapped back to OTU representative sequences to generate OTU abundance tables. Species taxonomic annotation was performed using mothur's classify.seqs command, primarily referencing the Silva 128 database with a confidence threshold of 0.6. Post-annotation, OTUs belonging to non-target domains (e.g., Archaea) were removed.

(4) Downstream Diversity and Differential Analysis： Alpha diversity was assessed using Chao1, ACE, Shannon, Simpson, and PD_whole_tree indices, supplemented by dilution curves and rank-abundance curves to validate sequencing depth. Beta diversity was calculated using Bray-Curtis, Jaccard distance, and Weighted/Unweighted UniFrac distances, visualized via Principal Coordinate Analysis (PCoA), Non-metric Multidimensional Scaling (NMDS), and clustering trees. Community structure was displayed through relative abundance bar charts and heatmaps at various taxonomic levels. Significant intergroup differences were validated using multiple statistical methods: Alpha diversity indices were analyzed via parametric tests (T-test/ANOVA) or nonparametric tests (Wilcoxon/Kruskal-Wallis); differences in community structure were assessed using ADONIS (PERMANOVA), ANOSIM, and MRPP; and key differential species or biomarkers were identified through LEfSe (LDA Effect Size) analysis.

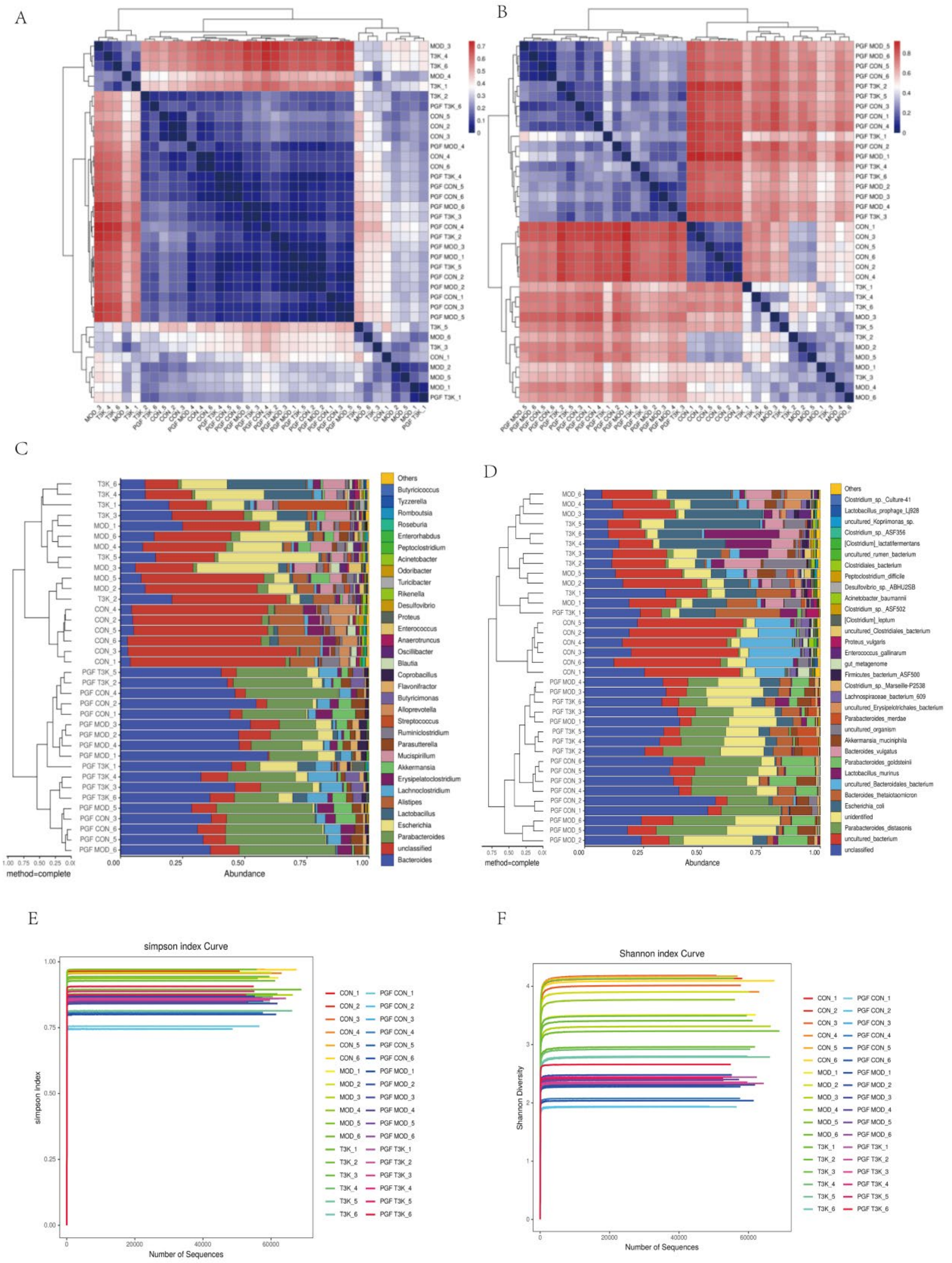

Supplementary Figure S4. A) Beta diversity heatmap at the genus level. B) Beta diversity heatmap at the species level. C) Combined analysis of species-level sample clustering tree and bar chart. D) Combined analysis of family-level sample clustering tree and bar chart. E) Simpson index plot. F) Shannon index plot.

Species	Strain	Resistant to acid	Resistant to bile	Adhesion/ Attachment	Antimicrobial	Immunomodulation	Antiproliferative	Antioxidant	PMID
Akkermansia muciniphila	ATCC BAA-835	√	√	∅	∅	√	×	√	36786638
Akkermansia muciniphila	DSM 22959	√	√	√	√	∅	∅	∅	32019075
Akkermansia muciniphila	AM01	√	√	∅	∅	∅	∅	∅	36786638
Akkermansia muciniphila	AM02	√	√	∅	∅	∅	∅	∅	36786638
Akkermansia muciniphila	AM03	√	√	∅	∅	∅	∅	∅	36786638
Akkermansia muciniphila	AM04	√	√	∅	∅	∅	∅	∅	36786638
Akkermansia muciniphila	AM05	√	√	∅	∅	∅	∅	∅	36786638
Akkermansia muciniphila	AM06	√	√	∅	∅	∅	∅	∅	36786638

Supplementary Table S1. Functional potential of multiple *Akkermansia muciniphila* strains. (Data source:https://probio-ichnos.streamlit.app/)
